# FERTILITY CARE IN LOW- AND MIDDLE- INCOME COUNTRIES: The future use of AI to improve accessibility of assisted reproductive technology in low- and middle-income countries

**DOI:** 10.1530/RAF-24-0077

**Published:** 2025-08-14

**Authors:** Gerardo Mendizabal-Ruiz, Omar Paredes, Ernesto Borrayo, Alejandro Chavez-Badiola

**Affiliations:** ^1^ Conceivable Life Sciences, New York, USA; ^2^ Laboratorio de sistemas autónomos para diseño biotecnológico, Departamento de Bioingeniería Traslacional, Universidad de Guadalajara, Guadalajara, Mexico; ^3^ Laboratorio de Innovación Biodigital, Departamento de Bioingeniería Traslacional, Universidad de Guadalajara, Guadalajara, Mexico; ^4^ IVF 2.0 Limited, London, United Kingdom; ^5^ Hope IVF Mexico, Mexico City, Mexico

**Keywords:** automation, *in vitro* fertilization, ART, artificial intelligence, standardization

## Abstract

**Abstract:**

People in low- and middle-income countries face many obstacles when trying to access fertility treatments. These challenges include high costs, the need for specialized medical facilities, and cultural beliefs that may discourage seeking help. This paper explores how artificial intelligence (AI) and automation could help overcome some of these barriers and make fertility treatments more widely available. It examines how AI may improve the accuracy, efficiency, and consistency of different steps in fertility treatments, such as choosing the healthiest embryos, analyzing sperm, evaluating eggs, and planning treatment. The paper also discusses how automation could simplify laboratory procedures, from growing embryos and freezing them for future use to the possibility of fully automating the *in vitro* fertilization (IVF) process, which could help lower costs and make these treatments more accessible. Finally, this paper addresses the ethical and practical challenges associated with using these technologies, including potential biases in AI, equitable access, quality control, data privacy, job implications, and cultural sensitivities.

**Lay summary:**

This paper explores how AI and automation could help make fertility treatments more accessible in low- and middle-income countries (LMICs). Many individuals and couples face difficulties conceiving, and assisted reproductive technology (ART) – which includes procedures such as intrauterine insemination (IUI) and IVF – offers them a chance to build a family. However, ART is often out of reach in LMICs due to high costs, the need for specialized medical facilities, and cultural barriers. AI and automation have the potential to improve accuracy, efficiency, and consistency in ART procedures, such as embryo selection, sperm and egg assessment, and treatment planning. Automation could also streamline laboratory processes, including embryo culture and freezing, which may eventually lead to more affordable and scalable fertility treatments. By reducing human error and dependence on highly trained specialists, AI-driven technologies could help lower costs and make ART available to more people. This paper also considers the ethical and practical challenges of using AI in reproductive medicine, including potential biases in AI algorithms, fairness in access to treatment, data privacy, workforce impact, and cultural sensitivities. Fertility treatments can be life-changing for those struggling with infertility due to medical conditions, age, or personal circumstances. They also provide opportunities for same-sex couples and individuals who want to start a family. However, in LMICs, infertility is often surrounded by social stigma, economic hardship, and limited medical resources, making access to ART even more difficult. By integrating AI and automation into reproductive medicine, it may be possible to break down these barriers, reduce costs, and create more inclusive and accessible fertility care. These advancements have the potential to bring hope to millions who dream of parenthood but currently lack the means to pursue it.

## Introduction

Assisted reproductive technology (ART) consists of a range of procedures and techniques aimed at assisting individuals or couples in achieving pregnancy. Two of the most widely used ART methods to generate fertilization are intrauterine insemination (IUI) and *in vitro* fertilization (IVF) (Matteo 2022).

IUI aims to increase the likelihood of pregnancy by directly depositing a concentrated sample of sperm into the woman’s uterus, maximizing the chances of successful fertilization within the fallopian tubes (Allahbadia 2017, Matteo 2022). The procedure typically takes only minutes and is performed in a doctor’s office or a fertility clinic. However, its effectiveness is inherently linked to various patient-specific factors, including age, underlying fertility issues, and the use of fertility drugs. Therefore, it may not be suitable for all individuals struggling with infertility.

IVF is a complex, multi-stage fertility treatment that takes place outside the body in a controlled environment to increase the probability of successful fertilization and pregnancy (Allersma *et al.* 2013, Matteo 2022). The process begins with ovarian stimulation to produce multiple eggs, followed by egg retrieval and laboratory fertilization using conventional methods or intracytoplasmic sperm injection (ICSI) (Sakkas & Gardner 2020, Matteo 2022).

After fertilization, the resulting embryos are monitored for several days to ensure proper development. Viable embryos are then evaluated, selected, and transferred into the female’s uterus using a thin catheter passed through the cervix. Any remaining embryos are cryopreserved (frozen) for future use. The current practice in many reproductive clinics is to opt for single embryo transfer. Typically, only one well-developed and high-quality embryo is chosen for transfer. However, there are situations where more than one embryo may be transferred. This decision is based on several factors, including the female’s age and health, the quality of the available embryos, and the recommendations of the medical team.

IVF offers several advantages for those facing infertility. It can help overcome issues such as low sperm count, poor egg quality, blocked fallopian tubes, or unexplained infertility (Szamatowicz & Szamatowicz 2020). In addition, IVF allows for genetic testing of embryos (preimplantation genetic diagnosis) to screen for certain inherited disorders before implantation (Kuliev & Rechitsky 2017, Traeger-Synodinos 2017).

The advancements in ART have had a profound impact on the lives of individuals and many couples facing fertility challenges. Its widespread adoption has led to a significant increase in the number of infants conceived through these methods. For example, in 2021 alone, ART accounted for 2.3% of all births in the United States (Sunderam *et al.* 2023). By offering solutions to overcome biological obstacles, ART can provide parents-to-be a sense of hope and the possibility of achieving their dreams of starting a family. The knowledge that options are available can be incredibly comforting and empowering, even for those who ultimately may not pursue or succeed with ART.

ART offers hope and possibilities for individuals who face the risk of infertility due to medical treatments or conditions. Cancer treatments like chemotherapy and radiation, while life-saving, can profoundly impact fertility by damaging reproductive organs, depleting ovarian reserves, or triggering premature menopause, leaving many survivors facing infertility or reduced fertility (Vakalopoulos *et al.* 2015, Salama & Woodruff 2017). As a result, many cancer survivors may face infertility or reduced fertility after completing their treatment. By preserving fertility through techniques such as egg freezing, embryo freezing, and ovarian tissue cryopreservation, ART allows these individuals to maintain the option of having biological children in the future. IVF is a viable choice if the patient has preserved eggs or embryos, allowing them to undergo the process to achieve pregnancy. If the patient’s eggs were not preserved or are no longer viable, using donated eggs or embryos to carry a pregnancy to term is another possibility. In situations where a patient is unable to become pregnant due to the removal of reproductive organs or other health issues, surrogacy offers an alternative path to parenthood. In this procedure, a gestational carrier (surrogate) is chosen to carry the pregnancy and give birth to the baby.

Women may choose to delay having children for various reasons, including establishing career stability and advancement, achieving financial security, navigating work-life balance challenges, pursuing personal and professional fulfillment, and coping with a lack of family-friendly workplace policies. Societal expectations and needs may lead women to prioritize their careers over motherhood in order to demonstrate their professional commitment and establish their credibility.

Delaying motherhood until later in life can come at the cost of fertility, as a woman’s reproductive potential naturally declines with age (Schmidt *et al.* 2011, Szalma & Djundeva 2020). The advent of ART, particularly egg freezing, has provided women with a tool to preserve their fertility while focusing on their careers.

ART has opened up new possibilities for the LGBTQ+ community to overcome biological barriers and fulfill their aspirations of parenthood. Through various techniques, ART allows same-sex couples and individuals to build their families, regardless of their sexual orientation or gender identity. For gay male couples, surrogacy using one partner’s sperm and a donor egg has become a viable option, allowing them to have a child biologically related to one partner. Reciprocal IVF offers lesbian couples a unique path to shared parenthood, allowing one partner to provide the eggs while the other carries the pregnancy. This approach fosters a profound biological connection between both parents and their children. Single women and lesbian couples can also use donor insemination to conceive, either through IUI or IVF. Transgender individuals who have undergone gender-affirming treatments can preserve their fertility through egg freezing, ensuring the possibility of having biological children in the future. In addition, embryo donation offers LGBTQ+ individuals the opportunity to experience pregnancy and childbirth, even if they cannot use their genetic material.

## ART in LMIC

Infertility is an important global health issue that affects millions of people around the world. According to the World Health Organization (WHO), around 10–15% of couples in the world suffer from infertility (Glujovsky *et al.* 2022). However, the societal impact of infertility in low- and middle-income countries (LMICs) is often more severe than in high-income countries due to various social, cultural, and economic factors. In many LMICs, having children is highly valued and often seen as a social necessity. Women, in particular, face immense pressure to bear children and are often blamed for infertility, even when the cause lies with their male partner. This situation can lead to social stigma, discrimination, and even violence against women who are unable to conceive. In some communities, infertility can result in divorce, abandonment, or ostracism, leaving women in a vulnerable and marginalized position (Rouchou 2013, Küçükkaya & Kiliç 2022).

The psychological impact of infertility can be severe, producing anxiety, stress, depression, and low self-esteem for individuals and couples (Küçükkaya & Kiliç 2022). The inability to fulfill societal expectations and the lack of support from family and community members can exacerbate these mental health challenges. Access to infertility diagnosis and treatment in LMICs is often limited, particularly for those living in rural areas or with lower socioeconomic status.

The societal impact of infertility in LMICs extends beyond the individual and family level. In countries where children are seen as a source of labor and financial security, infertility can have economic consequences, particularly for women who may be excluded from inheritance or property rights. Furthermore, in societies where having children is tied to social status and identity, infertility can have a profound impact on an individual or couple’s social standing within their community.

### Current barriers to accessing ART in LMIC

The high cost of ARTs, particularly IVF, poses a significant obstacle for many individuals and couples in LMICs who are struggling with infertility. The expense associated with ART can be attributed to several key factors, each contributing to the overall financial burden.

ART requires a highly specialized team of medical professionals, including reproductive endocrinologists, embryologists, andrologists, and nurses who have received extensive education and training to perform complex procedures such as egg retrieval, embryo transfer, ICSI, and embryo biopsy. The expertise of these professionals is essential for the success of ART, but it also comes with a high price. The high salaries paid to these specialists are reflected in the cost of treatment, as clinics must cover the expenses associated with maintaining a skilled medical team.

ART procedures require highly specialized laboratories with specific characteristics to ensure the safety, efficacy, and success of the treatments. An ART laboratory must be designed and maintained to prevent contamination from bacteria, fungi, and other microorganisms that can compromise the quality of the gametes and embryos. In addition to sterility, ART laboratories must maintain high air purity, as the presence of volatile organic compounds (e.g. benzene and formaldehyde) can harm embryo development (Sciorio *et al.* 2021). ART laboratories require specialized equipment for procedures, including high-quality microscopes essential for visualizing gametes and embryos, as well as micromanipulators that enable embryologists to perform delicate procedures on cells. Incubators with advanced monitoring systems are crucial for providing a controlled environment that mimics the human uterus, maintaining precise temperature, humidity, and gas levels to ensure optimal embryo development (Agarwal *et al.* 2022).

Moreover, ART laboratories require tools and consumables such as culture media, Petri dishes, and micropipettes. These materials must be of the highest quality and designed explicitly for ART procedures to ensure optimal results. The purchase, maintenance, and operation of this equipment and facilities are costly, and these expenses are passed on to the patients in the form of higher treatment fees.

Unfortunately, the success rates of ART procedures are not guaranteed, and patients may need to undergo multiple cycles of treatment before achieving a successful pregnancy. Each treatment cycle involves a significant investment of time, money, and emotional energy, and the cumulative cost of multiple cycles can be staggering. ART involves the use of expensive medications, such as hormones and other fertility drugs, which are necessary to stimulate ovulation, control the menstrual cycle, and prepare the uterus for implantation (Al-Inany *et al.* 2016). These medications represent a significant increase in the overall treatment cost, especially if multiple cycles are required.

The expenses associated with ART may be prohibitively expensive, especially in countries where the average income is low and health insurance coverage for infertility treatment is limited or non-existent. For many people in LMICs, the cost of a single IVF cycle can exceed their annual income, making it virtually impossible to access this type of care without incurring substantial financial debt.

Many LMICs do not have enough reproductive endocrinologists, embryologists, and other specialists trained to provide ART services. This shortage is especially severe in rural areas where healthcare services are limited. As a result, people and couples in these regions may need to travel long distances to get to fertility clinics, which can be time-consuming, expensive, and emotionally overwhelming.

Furthermore, the limited access to specialized facilities and equipment in LMICs can compromise the quality of infertility care. Many fertility clinics in LMICs may not have access to the latest technologies and equipment, such as high-quality embryology laboratories. These limitations can lead to a higher risk of complications, which can have serious health consequences for women undergoing treatment.

Addressing these barriers in LMICs requires a multi-faceted approach. Governments, healthcare organizations, and international agencies must collaborate to develop strategies that improve access to ART services. These strategies include initiatives such as subsidizing costs, training healthcare professionals, establishing specialized clinics, and promoting public-private partnerships. In addition, education and awareness campaigns are necessary to break down social stigmas, empower individuals to seek help, and foster a more supportive societal attitude toward infertility and ART.

## Artificial intelligence (AI) in ART

AI is a field of study within computer science that aims to develop electronic systems capable of performing tasks that usually require human-like intelligence. These tasks include abilities such as recognizing images and speech, making decisions, and understanding and translating languages. AI systems are designed to adapt to new information, learn from their experiences, and perform complex tasks by analyzing vast quantities of data and identifying relevant patterns (Soori *et al.* 2023).

AI and automation have the potential to enhance ART by improving efficiency, standardizing decision-making processes, and optimizing laboratory workflows. These technologies offer promising tools to support clinicians in treatment planning, patient monitoring, and outcome prediction, while also assisting embryologists in the lab with critical decision-making tasks such as oocyte evaluation, sperm analysis, and embryo selection (Bulletti *et al.* 2024, Wu *et al.* 2025, Zhang *et al.* 2025).

By processing large amounts of data from patient characteristics, treatment protocols, and outcomes, AI systems can be used to construct models that allow prediction of the optimal timing for oocyte retrieval and embryo transfer, potentially increasing the likelihood of successful fertilization and implantation (Asch Schuff *et al.* 2024, Lee *et al.* 2024). These predictions help clinicians personalize treatment plans, adjust medication dosages, and make informed decisions at various stages of the ART process. AI tools can analyze millions of data points, considering factors such as patient characteristics, embryo morphology, and lab conditions to predict the likelihood of a successful outcome (Fernandez *et al.* 2020, Hanassab *et al.* 2024).

Thyroid disorders are linked to reduced fertility and pregnancy complications, especially during controlled ovarian hyperstimulation in ART (Krassas *et al.* 2010). The integration of AI in thyroidology has been suggested as a potential means to enhance diagnosis, treatment, and patient management across various thyroid disorders, including thyroid nodules, thyroid cancer, and autoimmune or functional thyroid diseases (Toro-Tobon *et al.* 2023, Yang *et al.* 2024).

Vitamin D deficiency and other micronutrient shortfalls correlate with lower ART success rates (Wang *et al.* 2025). Machine-learning models that incorporate serum 25-hydroxyvitamin D levels into standard clinical factors enhance outcome prediction (Jiang *et al.* 2025), underscoring nutrition as a modifiable factor in infertility care. Advances in nutrigenomics and nutri(epi)genetics reveal genotype-specific nutrient requirements, allowing for tailored diets and targeted nutraceuticals to modulate reproductive pathways (Kohil *et al.* 2022). AI-driven personalized-nutrition platforms are poised to translate multi-omics and lifestyle data into patient-specific prescriptions that complement ART and boost pregnancy rates (Armand *et al.* 2024).

Monitoring ovarian follicle growth with transvaginal ultrasound (TVUS) is crucial for obtaining viable eggs in IVF, but it is a time-intensive process. AI-driven 3D ultrasound segmentation presents a promising solution by reducing dependence on operator expertise and enhancing efficiency (Li *et al.* 2019, Yang *et al.* 2021). AI-driven models that integrate ultrasound imaging with clinical parameters have been explored to enhance the assessment of endometrial receptivity and, consequently, predict clinical pregnancy (Liang *et al.* 2023).

The performance of an embryologist in making critical decisions during an IVF treatment is influenced by a complex interplay of factors, including their level of training, years of experience, and personal characteristics. While an embryologist’s expertise can significantly contribute to the procedure’s success (Alteri & Koustas 2024), the inherent subjectivity in their decision-making process can introduce variability in the treatment outcomes. This variability can be further compounded by human factors such as fatigue, emotional state, and circadian rhythms, which affect an embryologist’s judgment at different times of the day or week (Murphy *et al.* 2024). Consequently, the IVF process is not entirely objective or data-driven, and the success rates may fluctuate depending on the embryologist’s performance (Tiegs & Scott 2020, Cirillo *et al.* 2022). AI could help overcome these challenges by enhancing embryologists’ capabilities through real-time data analysis and pattern recognition, thereby supporting better-informed decisions (Zaninovic & Rosenwaks 2020, Salih *et al.* 2023). By standardizing the decision-making process across embryologists and clinics, AI promises to reduce variability in treatment outcomes (Sharma & Khan 2024), leading to more consistent and optimized fertility treatments (Rosenwaks 2020).

Oocyte assessment involves evaluating morphological features such as the size and shape of the cell, the presence of any abnormalities or inclusions, and the characteristics of the surrounding zona pellucida and polar body (Rienzi *et al.* 2023). AI algorithms are trained using datasets of oocyte images with known outcomes, allowing them to learn the morphological characteristics associated with successful fertilization and embryo development. Once trained, these algorithms can be used to predict the viability of new oocyte images with high accuracy, providing a more objective and standardized approach to oocyte assessment compared to manual evaluation (Fjeldstad *et al.* 2024).

Traditional methods of sperm evaluation rely on manual microscopic assessment of parameters such as count, morphology, and motility, which can be subjective and time-consuming (Dias *et al.* 2019). In contrast, AI-powered systems can quickly analyze thousands of sperm, providing a more precise and objective evaluation. For example, computer-assisted semen analysis systems utilize AI to provide standardized and efficient assessments of sperm concentration, motility, and morphology, thereby reducing the inconsistency and subjectivity associated with manual evaluation (Valverde *et al.* 2020). AI apps such as Mojo and Yo allow men to test their sperm count at home, breaking down barriers such as cost and stigma associated with seeing a fertility doctor, and empowering men to take charge of their reproductive health. AI has also been used to predict sperm DNA fragmentation index, a key factor impacting fertility, as high DNA fragmentation can lead to poor fertilization, low embryo quality, and a higher risk of miscarriage (Haddock *et al.* 2021, Kumar *et al.* 2023).

Furthermore, AI sperm selection tools such as SiD utilize computer vision to automatically detect, track, and rank sperm in real-time based on the microscope video feed. Higher SiD scores correlate with successful fertilization and blastocyst formation (Mendizabal-Ruiz *et al.* 2022). For procedures such as ICSI, AI sperm selection tools can assist embryologists in identifying the optimal sperm for injection, thereby enhancing the chances of successful fertilization (Montjean *et al.* 2024).

Embryo selection is a critical step in IVF, as it directly impacts the success rates of implantation and live births. Traditionally, embryologists assess embryo quality based on morphological characteristics observed under a microscope (Alpha Scientists in Reproductive Medicine and ESHRE Special Interest Group of Embryology 2011). However, this manual method is subjective, time-consuming, and prone to inter-observer variability. With the integration of AI algorithms such as computer vision and machine learning, it is possible to provide objective, consistent, and rapid embryo quality assessment, assisting embryologists in selecting the most viable embryos for transfer. AI systems can analyze images of embryos and identify morphological features that correlate to successful implantation and live birth. These features may include cell number, symmetry, fragmentation, and texture (Louis *et al.* 2021). AI algorithms are trained on large datasets of embryo images with known outcomes, allowing them to learn patterns and associations that may not be apparent to human observers. AI models such as the Embryo Ranking Intelligent Classification Algorithm (ERICA) (Chavez-Badiola *et al.* 2020) and STORK-A (Barnes *et al.* 2023) utilize morphological features to predict embryo ploidy and implantation potential, providing a standardized approach to embryo selection. Time-lapse imaging combined with AI provides a comprehensive view of embryo development, potentially leading to more accurate predictions of pregnancy success. Helping embryologists select the most viable embryos for transfer could improve IVF success rates and reduce the need for multiple cycles. AI-assisted embryo selection has been widely used in many labs with promising results (Zaninovic & Rosenwaks 2020). However, it is important to note that, to date, a clinical trial has not conclusively shown that AI outperforms experienced embryologists in improving pregnancy rates (Illingworth *et al.* 2024). This result highlights the need for further validation before AI can be considered a definitive improvement over human expertise.

Ovarian aging and dysfunction are significant barriers to fertility, particularly for women with premature ovarian insufficiency, diminished ovarian reserve, or age-related infertility. As ovarian function declines, the quantity and quality of eggs decrease, reducing the chances of conception. Stem cell therapy has emerged as a promising regenerative approach to restore ovarian function and potentially enhance fertility outcomes (Kim & Kim 2024). The combination of AI, gene editing, and stem cell-based therapy promises to make precise cell therapy a promising approach for rescuing female reproductive health (Wang *et al.* 2024*b*).

The ability to generate functional oocytes from stem cells represents a groundbreaking advancement in reproductive medicine. This innovation offers hope for individuals with infertility due to ovarian failure, genetic conditions, or age-related decline. By harnessing stem cell technologies, researchers are working to create lab-generated eggs that could one day be used in reproductive medicine (Grettka *et al.* 2024, Aizawa *et al.* 2025). Stem cell research may greatly benefit from AI’s capability to analyze large-scale data, enabling faster, more precise insights into cell differentiation, regenerative potential, and therapeutic applications (Kim & Hong 2024).

## Automation in ART

Automation involves using technology to perform tasks that typically require human intervention, with minimal or no direct human control. Automation integration in ART involves utilizing various technologies, including AI, computer vision, time-lapse imaging, robotic controllers, and electronic medical records, to streamline and optimize different aspects of the fertility treatment process. The integration of automation in ART has the potential to revolutionize fertility treatments by enhancing efficiency and accuracy.

One of the most promising aspects of automation in ART is its potential to standardize various processes within the IVF lab, which can lead to improved success rates, reduced variability in treatment outcomes, and increased efficiency. By reducing subjectivity in decision-making processes, optimizing treatment protocols, and personalizing care, these technologies may significantly improve the time to pregnancy for couples struggling with infertility and reduce the cost of IVF treatments.

Autonomous devices, such as robotic culture and embryo storage systems, are currently used in many IVF labs worldwide. Robotic time-lapse incubators are an innovative technology that combines the benefits of time-lapse imaging with the precision and consistency of robotics to provide a stable, controlled environment for embryo development while continuously monitoring and documenting their growth through high-resolution imaging. The robotic system enables imaging of embryos without removing them from the incubator, thereby minimizing exposure to temperature, humidity, or gas composition fluctuations that can negatively impact their development. These systems ensure consistent and reproducible culture conditions across different patients and IVF cycles, which may help reduce variability in treatment outcomes and facilitate data comparison across different clinics or research studies. Moreover, the large amount of standardized data generated by these systems enables advanced AI algorithms to analyze embryo development patterns and try to predict their implantation potential. Examples of commercially available robotic time-lapse incubators include the Geri (Genea Biomedx, Australia), EmbryoScope+ (Vitrolife, Sweden), and MIRI TL (Esco Medical, Singapore).

Robotic cryo storage systems are an advanced technology for cryopreservation and managing embryos in IVF laboratories. These systems can automatically identify, track, and retrieve individual embryo samples using barcodes or radio-frequency identification (RFID) tags, thereby reducing the risk of human error and ensuring accurate correspondence between retrieved embryos and patients. Robotic storage systems minimize the need for manual handling of embryos, reducing the risk of contamination or damage. Access to the stored embryos can be restricted and monitored through electronic logs, enhancing the security of the samples. These systems may be integrated with electronic medical records and laboratory information management systems. This integration allows for seamless data transfer, tracking, and documentation of stored embryos. The TMRW is an example of a commercially available system.

Full IVF lab automation has the potential to increase the scalability of ART by reducing the need for a large number of highly qualified personnel to perform fertility treatments. A technician could operate the machines in a fully automated IVF lab, while an embryologist would only be required to handle complex cases or address equipment malfunctions. This streamlined staffing approach helps to optimize efficiency and productivity, and it could significantly reduce the cost of fertility treatments, increasing accessibility for more patients.

Automating the whole IVF procedure pipeline involves building robotic systems capable of performing the different tasks that embryologists currently carry out in the lab, including preparing dishes and reagents. These systems must also be capable of accurately identifying gametes and embryos and handling them delicately to perform egg finding and denuding, sperm preparation, insemination, culture, vitrification, and storage.

Procedures such as egg retrieval and denudation require the system to locate all the cumulus–oocyte complexes (COCs) present in a Petri dish containing follicular fluid, and then use a pipette to absorb, isolate, and clean them. Denudation of eggs requires the ability to expose the COC to hyaluronidase and then use mechanical force to strip the cumulus and corona cells without damaging the egg (Weng *et al.* 2018). Moreover, an AI is needed to determine when to stop the stripping process. Current approaches to automating COC denuding include the use of microfluidics (Weng 2019), ultrasound (Mokhtare *et al.* 2022, Sánchez *et al.* 2020), as well as computer vision and robotics (Zhai *et al.* 2023).

Sperm preparation involves selecting the healthiest and most viable sperm for fertilization. This procedure requires liquid handling devices to process the raw ejaculate using a protocol to separate the motile, morphologically normal sperm from the seminal plasma, which contains debris, dead sperm, and other cells that can interfere with fertilization. Examples of methods for automatic sperm preparation are primarily based on microfluidics (Xiao *et al.* 2021, Chinnasamy *et al.* 2017, Olatunji & More 2022).

An autonomous insemination system using ICSI requires AI to identify and evaluate the characteristics of single spermatozoa in a PVP droplet, enabling them to be immobilized and loaded into a needle for use. Then, the system must be able to locate an egg in an independent droplet, identify its morphological characteristics, and position the holding pipette and the needle to hold it and inject it without damaging it. Examples of automation approaches for ICSI include the robotic immobilization of sperm (Zhang *et al.* 2019), the robotic injection of sperm into the oocyte (Costa-Borges *et al.* 2023), and a complete automation system that enables the procedure to be performed remotely (Mendizabal-Ruiz *et al.* 2025).

Vitrification requires exposing the gamete or embryo to cryoprotectant solutions for a predetermined amount of time, then placing the cell into a cryo device and submerging it in liquid nitrogen (LN2) as quickly as possible to avoid crystal formation. An autonomous system could help alleviate the challenges related to standardization and the time required to perform this procedure (Arav & Patrizio 2024). Current approaches for automation of vitrification rely on cell preparation using microfluidics (Hajek *et al.* 2021, Miao *et al.* 2022, Wang *et al.* 2024*a*). Moreover, robotic manipulators could give a unique opportunity to perform the LN2 plunging with a speed and precision level that is impossible for a human.

By leveraging the vast amounts of standardized data generated during each IVF cycle using a fully autonomous system, AI can recognize patterns that may not be obvious to human experts. These patterns would lead to the development of more advanced and personalized treatment protocols that take into account the unique characteristics of each gamete or embryo, as well as their developmental progress. By personalizing IVF treatments at the cellular level, clinics can further optimize the chances of success for each patient (Hanassab *et al.* 2024).

By automating repetitive and time-consuming tasks, robotic controllers may help alleviate the workload on embryologists and other lab staff, allowing them to focus on more complex aspects of patient care. This level of precise control could maximize the potential of each IVF cycle, increasing the chances of successful fertilization, embryo development, and implantation, and reducing the cost of IVF treatments, as the need for highly trained personnel on-site may decrease.

## Ethical considerations and challenges

While AI and automation can potentially increase access to fertility treatments by reducing costs and the need for highly skilled personnel, it is crucial to ensure that they are implemented in a safe, equitable, and ethically responsible manner.

As most AI systems are based on learning using existing data, it is essential to consider the potential for bias due to differences in population characteristics between the countries where the AI models are trained and where they are applied. For example, environmental factors such as pollution exposure and lifestyle habits can vary significantly between high-income and medium- and low-income countries. These factors can impact fertility, embryo development, and pregnancy outcomes. AI models trained on data from populations with better nutrition, lower exposure to pollutants, and healthier lifestyles may not accurately predict outcomes in populations facing different environmental challenges. Moreover, socioeconomic factors such as access to healthcare, education, and financial resources can influence fertility and pregnancy outcomes. AI models trained on data from populations with better access to these resources may not accurately predict outcomes in populations facing more significant socioeconomic barriers. Therefore, developing AI models using diverse and representative datasets that include data from various populations, including those in medium- and low-income countries, is crucial. In addition, it is essential to validate AI models in the specific populations where they will be applied, ensuring they are accurate and effective in those contexts. Local validation studies can help to identify any biases or limitations in the AI models and guide necessary adjustments or adaptations.

Although automation may lower the cost of ART, policymakers and healthcare providers must collaborate to develop strategies that promote equitable access to these technologies, such as subsidies, insurance coverage, or public-private partnerships. This collaboration is particularly essential in LMICs, where socioeconomic disparities are often more pronounced.

Quality control and regulation are also critical considerations. Automated ART systems must be subject to rigorous quality control measures and regulatory oversight to ensure their safety, efficacy, and reliability. Regulatory bodies in these countries may need to develop new guidelines and standards specific to automated ART technologies, considering factors such as infrastructure, technical expertise, and cultural context.

Data privacy and security are also crucial, as automated ART systems generate and rely on vast amounts of sensitive patient data. Robust data privacy and security measures must be in place to protect this information from unauthorized access, breaches, or misuse. Medium- and low-income countries may need to invest in secure data infrastructure and develop comprehensive data protection regulations to safeguard patient privacy.

The introduction of automation in ART may also have significant implications for the healthcare workforce in these countries. While automation may help address shortages of skilled personnel, it may also lead to job displacements or require extensive retraining of existing staff. Policymakers and healthcare institutions must develop strategies to manage these workforce transitions, such as providing education and training opportunities, creating new roles for displaced workers, and ensuring fair labor practices.

Cultural and religious considerations may also come into play, as the use of ART, whether automated or not, may raise concerns in some communities. Policymakers and healthcare providers must be sensitive to these concerns and engage in open dialog with community leaders and religious authorities to address ethical or moral objections. Public education campaigns can help raise awareness about the benefits of ART and dispel misconceptions or stigmas associated with fertility treatments.

## Conclusion

AI and automation could reshape ART in LMICs. ML models deliver standardized, bias-free evaluation of gametes and embryos, while robotic platforms automate micromanipulation, incubation, and quality control. In theory, these tools shorten procedure times, reduce dependence on scarce expert embryologists, and pave the way for fully robotic IVF suites capable of high-throughput, reproducible care.

Evidence, however, is still limited. Large double-blind trials have not shown AI embryo selection to outperform experienced embryologists, and robust multi-center data on durable cost savings are lacking. Steep capital outlays, regulatory approvals, cybersecurity safeguards, and staff retraining can raise costs before efficiencies emerge.

Democratizing ART, therefore, hinges on more than technology. Supportive regulation, innovative financing and insurance models, reliable power and data infrastructure, and culturally sensitive patient engagement are critical. Importing sophisticated hardware, complying with privacy legislation, and securing maintenance contracts can create fresh logistical and financial barriers, particularly in resource-constrained health systems.

Future research should pair technical validation with economic modeling, health system capacity analyses, and ethics. They must verify whether AI tools are affordable, whether clinics can actually use them, and whether they meet ethical standards.

AI and automation are potent enablers but not silver bullets. When deployed responsibly and contextually, they can move ART toward greater availability and affordability; without such coordination, they risk widening, rather than closing, existing access gaps.

## Declaration of interest

ACB and GM are shareholders at Conceivable Life Sciences and IVF2.0. OP is an employee at IVF2.0.

## Funding

This research did not receive a specific grant from any funding agency in the public, commercial, or not-for-profit sectors.

## Author contribution statement

GM and ACB participated in planning, conducting a comprehensive literature review, organizing the structure, and contributing to the writing and editing of the manuscript. OP and EB conducted extensive research, critically analyzed the literature, and actively contributed to the writing process, including drafting sections and providing valuable insights during the revision process.
